# Mobile telephone apps in mental health practice: uses, opportunities and challenges

**DOI:** 10.1192/pb.bp.114.050005

**Published:** 2015-12

**Authors:** Justin Marley, Saeed Farooq

**Affiliations:** 1 North Essex Partnership University Foundation Trust; 2 Black Country Partnership NHS Foundation Trust

## Abstract

Smartphones are used by patients and clinicians alike. Vast numbers of software applications (apps) run on smartphones and carry out useful functions. Clinician- and patient-oriented mental health apps have been developed. In this article, we provide an overview of apps that are relevant for mental health. We look at clinician-oriented apps that support assessment, diagnosis and treatment as well as patient-oriented apps that support education and self-management. We conclude by looking at the challenges that apps pose with a discussion of possible solutions.

Mobile telephones are ubiquitous and have more recently evolved into the smartphone, a combination of a handheld computer and mobile telephone. Smartphones support software applications (apps) with specialised functions. Some apps may be pre-installed on the telephone but many apps are downloaded. Downloading apps (either free or for a nominal fee) is straightforward. Apps are available for a large number of mental health conditions and are also known as mHealth apps. Many of these apps have already been investigated by researchers and include apps for psychosis,^1^ depression,^2^ anxiety,^3^ alcohol use disorders,^4^ smoking cessation,^5^ sleep disturbance^6^ and weight loss.^7^ In this article we will avoid the more generic productivity apps (e.g. email apps) and will instead focus on general clinical and mHealth apps as well as regulated apps where possible.

## Clinician-oriented apps

### Assessment and diagnosis

Smartphone apps can aid in diagnosis in two main ways: by supplying clinicians with diagnosis-related information and less commonly through data acquisition. Apps can be used in clinical encounters or remotely, although the latter overlaps with self-monitoring. Apps have proven useful in assessment and diagnosis in clinical practice. In one study, 50% of doctors reported that apps facilitated diagnosis during on-call work whereas 43% reported apps helped them interpret laboratory values.^8^

**Fig. 1:**
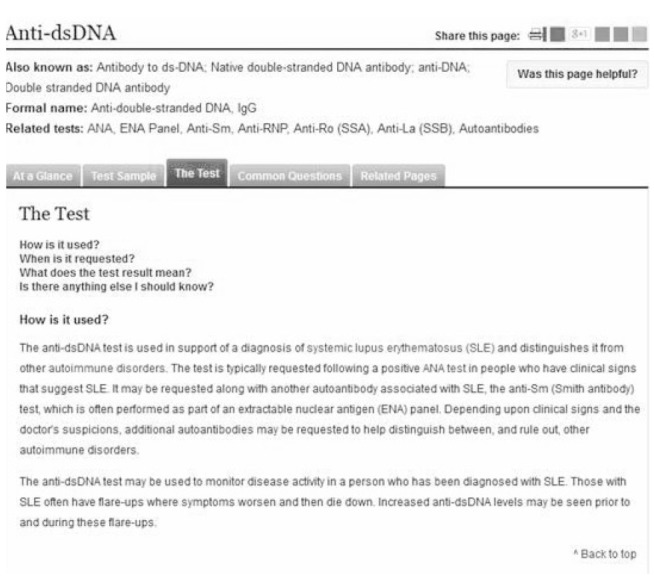
Screenshot from the desktop version of Lab Tests Online-UK

As an example, Lab Tests Online UK (www.labtestsonline.org.uk) is a peer-reviewed online resource for clinical laboratory tests with corresponding apps for the iPhone and Android (Fig. 1). The app enables the clinician to look up explanations of test results through a search facility or an index. The explanations are intended for the general public but there is enough information there to be useful for clinicians. Other useful features include news updates, information on screening for populations and an index of conditions.

There are a large number of apps relating to the diagnosis. Many of these are American apps that supply ICD-10 coding information for billing purposes. There are a number of variations including apps that convert between ICD-9 and ICD-10. The American Psychiatric Association has produced a DSM-5 app that contains detailed information on the DSM-5 diagnostic categories as well as ICD-10 coding equivalents. The app includes a series of instructive videos on many of the diagnostic categories.

Less commonly, apps can also be used in conjunction with hardware devices that connect to the smartphone. These apps may acquire and process data from external appliances such as digital stethoscopes, ophthalmoscopes and electrocardiogram (ECG) monitors. A similar principle can be seen with the self-monitoring apps that can provide clinicians with patient-collected data. Storing patient data on mobile apps brings additional complications relating to the Data Protection Act 1998 as well as principles of patient confidentiality that need to be worked through appropriately.

### Treatment

Treatment-related mHealth apps can be broadly divided into medical and psychosocial and may be professional or patient-focused.

### Medical apps

In terms of UK practice, the National Institute for Health and Care Excellence (NICE) produces both the *British National Formulary* (BNF) app and an app for NICE guidance (www.nice.org.uk/about/what-we-do/nice-apps-for-smartphones-and-tablets). The NICE BNF app offers a search facility to rapidly locate drug information and contains the contents of the paper version of the BNF. The app ensures that clinicians are able to access an up-to-date BNF even in the busiest clinical environments. This is contingent on the accessibility of the smartphone as well as regular updating of the app. The NICE guidance app features indexed evidence-based guidance from NICE on the management of a range of conditions. This app effectively utilises the large storage capacity of current smartphones.

### Apps to assist in psychosocial interventions

There are many psychology-related apps, including those listed on the NHS Choices apps site (http://apps.nhs.uk) such as Phobia Free. Phobia Free supports the use of exposure therapy for the treatment of phobias in the form of games and augmented reality. Rizvi *et al* described an app called DBT Field Coach that provided instructions, exercises and reminders to help patients with borderline personality disorder manage emotional crises.^9^ The researchers found that the app helped patients to self-monitor and complete homework assignments.

Another group of apps support patient education. One example is the Brain Tutor 3-D/HD (online Fig. DS1) which features a 3-D magnetic resonance imaging (MRI) reconstruction of a brain (www.brainvoyager.com/Mobile/BrainTutorHD_iOS.html). The clinician is able to select views of a virtual brain or an MRI reconstruction of the head including the brain. All views support 3-D manipulation of the images. For instance, the clinician may explain the MRI findings to a patient and then proceed to demonstrate these on the 3-D models on their smartphone.

## Patient-oriented apps: education and self-management

A large proportion of mental health apps are directed towards the public. One example of this is Moodscope from NHS Choices (http://apps.nhs.uk/), which enables a person to track their mood. The scores can be stored and a patient may allow the clinician to access their records to facilitate assessment. SAM (Self-Help Anxiety Management) supports patients in managing panic attacks. These apps support self-management of conditions including chronic illnesses through diary functions and education. Such apps may be particularly useful after discharge from specialist services or in-between appointments.

Self-monitoring apps create personal health records which are fundamentally different from clinical patient records in their function and composition. Patients will be more empowered by holding their own records and managing access rights to professionals. However, this is accompanied by a variability in the quality of information held in the records,^10^ an expanding number of record systems and the potential for further use of these records by third parties. The divergence of app-based personal health records and clinical records will generate complex interactions between these two systems.

## Challenges

Smartphone apps present many challenges (Box 1). One of the primary difficulties is the regulation of mental health apps due to their abundance. A number of studies have highlighted evidence of unsafe medical apps^10^ and the US Food and Drug Administration (FDA), the UK Medicines and Healthcare Products Regulatory Agency (MHRA) and NHS England have started to regulate apps. The NHS Choices website identifies regulated clinical apps and lists a number of other medication-related apps intended for professionals and patients. The relevant regulator depends on the function of the app. For instance, the MHRA would regulate apps classed as medical devices.

Self-certification has been suggested as one solution for the regulatory challenges.^11^ Lewis & Wyatt suggest a regulatory framework which addresses challenges intrinsic and external (e.g. hardware) to the app.^10^ Charani and colleagues go one step further, arguing that there needs to be a governance and legal framework in place for the use of apps in clinical practice.^12^ If clinicians or subject matter experts have not been involved in app development this may influence its quality and effectiveness. An absent evidence base for an app may limit clinical uptake. Privacy and security are other challenges for the app market.

The effectiveness of apps may also depend on the characteristics of the patient population. In one study looking at smartphone apps for weight loss, young adults considered simple weight measurement alone too narrow in focus and advocated behavioural software features.^13^ Access to a smartphone was negatively correlated with age in one study looking at consecutive patients in a neuropsychiatry and memory clinic.^14^

**Box 1** Challenges of mental health apps

Extrinsic to the app:

- hardware constraints that limit the apps.^15^

Intrinsic to the app

Relating to the patient or app user:

- concerns about how the data will be used by the app service.

Relating to the clinician:

- accuracy of clinically related processes^16^
- lack of medical involvement in app development^17^
- insufficient information to keep doctors or medical students interested.^18^

Relating to the healthcare service:

- deviation from or lack of evidence based practice recommendations^3^
- patient confidentiality issues
- clinical risk emerging from use of apps.^19^

## Conclusions

Mental health apps have expanded rapidly in number and regulation is playing catch-up. There are many useful apps that can support clinicians in the assessment and management of patients. There is also a burgeoning market of personal health apps that are reshaping the health economy. A sustained and multifaceted response from individual clinicians, health services and policy drivers is needed to adapt to this new health economy.
